# A comparative study of the diagnostic accuracy of cone beam computed tomography and intraoral radiographic modalities for the detection of noncavitated caries

**DOI:** 10.1007/s00784-014-1282-6

**Published:** 2014-07-25

**Authors:** J. Krzyżostaniak, T. Kulczyk, B. Czarnecka, A. Surdacka

**Affiliations:** 1 Department of Conservative Dentistry and Periodontology, Poznan University of Medical Sciences, Poznań, Poland; 2 Department of Biomaterials and Experimental Dentistry with Section of Dental Radiology, Poznan University of Medical Sciences, Poznań, Poland

**Keywords:** Cone beam computed tomography, Radiography, Dental caries

## Abstract

**Objectives:**

The aim of this study was to determine the diagnostic accuracy of cone beam computed tomography (CBCT) in the detection of approximal and occlusal noncavitated carious lesions.

**Methods:**

A total of 135 extracted human posterior teeth were used. They were radiographed using the following: conventional film (Kodak Insight), a digital system (PSP plates Digora Optime, Soredex), and a CBCT system (NewTom 3G, Quantitative Radiology). All the images were assessed by two independent observers twice. Receiver operating characteristic analysis (Az) was used.

**Results:**

NewTom 3G 9″ CBCT was statistically inferior to conventional film and a digital system for the detection of approximal caries. NewTom 3G 9″ had significantly higher Az values than PSP plate (*p* < 0.05), but there were no statistically significant differences between the Az values for CBCT and film (*p* > 0.33) for occlusal surface caries.

**Conclusion:**

The diagnostic accuracy of all three tested radiographic systems is low, and it is necessary to use other methods to improve early caries detection.

**Clinical relevance:**

CBCT has found a wide application in different fields of dentistry. The report from a CBCT examination performed for any of these reasons should include all abnormal findings, and the detection of noncavitated caries lesions is especially important because it facilitates the use of noninvasive treatment.

## Introduction

There are two main contemporary treatment approaches regarding caries: preventive (PCA—preventive care advised) and operative (OCA—operative care advised). Both are now routinely used for caries management in clinical practice [1]. Since the operative treatment of advanced dental caries will probably not reduce the incidence of this disease significantly, efforts should be focused on its early detection and the use of treatment strategies which can preserve the intact status of the tooth surface. Therefore, prophylaxis and noninvasive methods of treatment are the desired aims. The need for early detection of noncavitated carious lesions has been emphasized in the literature repeatedly [2]. However, a single diagnostic tool which is noninvasive and which is capable of providing a simple, reliable, sensitive, and specific measurement of the size of carious lesions does not exist [3].

The specific anatomical relations of the proximal surfaces and the occlusal surfaces of the tooth produce different manifestations of the carious process. An accurate detection of the disease poses different problems. Interproximal caries develops between two contacting surfaces of adjacent teeth whereas occlusal caries begins and develops on the cuspal slopes and at the bottom of the fissures. In both types of lesions, direct visual and tactile examinations are hindered. A number of modern methods for detecting caries have been developed. These are based on visual or laser light, electrical current, and ultrasound, but intraoral radiographic examination is still most commonly used in routine dental practice [3, 4]. Both conventional dental films and digital detectors are used as either solid-state detectors or photostimulable phosphor plates (PSP) [5, 6]. These intraoral systems provide two-dimensional information about a tooth and the neighboring mineralized structures and are usually valuable for the detection of proximal caries. However, for the detection of occlusal caries, the value of radiographic examination as the main additional test is controversial, especially in early stages of the disease.

In recent years, cone beam computed tomography (CBCT) has become more widely used in dentomaxillofacial imaging [7, 8]. CBCT provides three-dimensional images and supplies sagittal, coronal, and axial images plus their multiplanar transformation. Background noise from CBCT images can be eliminated. The method is now being used in several dental fields including surgery and traumatology [9], implantology [10], orthodontics [11], endodontics [12], periodontology [13], and the diagnostics of temporomandibular joint diseases [14]. Previous data from the literature regarding both approximal caries [15–23] and occlusal carious lesions [17, 18, 23–25] are controversial. There has been one study [17] in which NewTom 3G system was used in evaluation of proximal caries detection.

Based on a systematic literature review, guidelines for the application of CBCT have been elaborated by the SEDENTEXCT project in Europe [http://www.sedentexct.eu/files/radiation_protection_172.pdf]. These guidelines do not recommend CBCT for caries diagnosis mainly because of the higher radiation dose involved compared to that form of intraoral radiography.

The aims of the present study were to determine the diagnostic accuracy of CBCT in the detection of approximal and occlusal noncavitated, carious lesions and to compare this accuracy with that from two intraoral modalities: the *F* speed film and the PSP plate.

## Methodology

The study was based on 135 noncavitated extracted human teeth: 67 premolars and 68 molars. Only teeth with macroscopically intact occlusal and approximal surfaces were qualified for investigation. Teeth with loss of tissue and teeth with fillings were excluded. After extraction, the teeth were cleaned with a cotton gauze pad and stored in 10 % formalin solution. The collection time was about approximately 6 months. The teeth were randomly placed in silicon blocks with approximal contacts, four in a row: two premolars and two molars. The teeth were placed in the molds with their most prominent convexities forming the contact point with the neighboring, thus simulating the normal situation in the mouth.

All the blocks were imaged, using two intraoral systems (conventional film and a digital system) and the CBCT system as follows:

1. Standard radiographs were obtained using Kodak Insight film (Eastman Kodak Company, Rochester, NY) with an image exposure time of 0.25 s. These intraoral radiographs were exposed using standardized conditions: 70 kVp, 8 mA, 32-cm focus-tooth distance, 1-cm tooth-receptor distance, and the paralleling technique. The films were developed in an automatic roller processor (XR4pro Dürr Dental).
2. Digital radiographs were obtained using an intraoral photostimulable phosphor plate system (Digora Optime, Soredex, Helsinki, Finland). The same intraoral X-ray unit as above was used, and it was operated under the same standardized conditions with an image exposure time of 0.11 s. The plate was scanned in a Digora Optime scanner.
3. The CBCT data were acquired from the NewTom 3G system (Quantitative Radiology, Verona, Italy) in an FOV of 9 in (voxel size 0.25, medium resolution). The images were obtained at 110 kVp, automated adjusted milliamperes, and a total scan time of 36 s. The images obtained from the CBCT system were reconstructed with proprietary software and sectioned in the mesiodistal plane (imaging layer thickness 0.25 mm).

Next, two independent observers assessed the obtained images twice, with a 2-week interval. They examined the occlusal and approximal surfaces of all the teeth. A 5-point rating scale was used to assess the presence and extent of primary occlusal and approximal carious lesions in each tooth as follows: 0, sound (no caries detected); 1, radiolucency in the outer half of the enamel; 2, radiolucency in the inner half of the enamel reaching, but not crossing, the amelo-dentinal junction; 3, radiolucency in the outer third of the dentin; and 4, radiolucency reaching deeper into the dentin. Each observer evaluated the conventional radiographic films on a light box with a × 5 magnifier if necessary. The digital images were displayed on a 19″ monitor. The observers separately assessed all the images with the same PC located in the same dimmed room. The images from the digital system and the CBCT system were examined using proprietary software (Digora for Windows and NewTom station v.2.04, respectively) both of which allowed the operators to use image enhancement facilities.

**Fig. 1:**
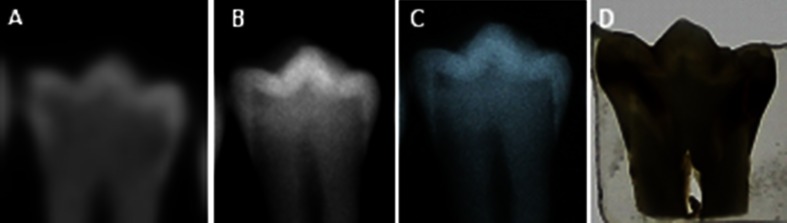
Images of approximal caries obtained by with CBCT (**a**), storage phosphor plate digital system (**b**), conventional radiography (**c**), and histologic section (**d**) of the same tooth

**Fig. 2:**
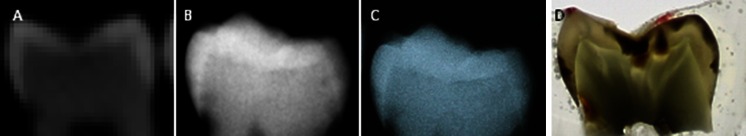
Images of occlusal caries obtained with CBCT (**a**), storage phosphor plate digital system (**b**), conventional radiography (**c**), and histologic section (**d**) of the same tooth

After the radiographic imaging, a histological investigation was carried out as a reference test (“gold standard”) in order to conduct a true assessment of the extent of any carious lesions present. The histological observation served as the validating criteria for the presence and depth of carious lesions. Each tooth was embedded in acrylic (Duracryl Plus Polymer, Spofa Dental) and sectioned into 700-μm-thick sections in the mesiodistal plane, using a 100-μm diamond band. The sections were attached to a microscope slide with transparent varnish. Two experienced observers (different from those evaluating the radiographic images) assessed the tooth sections using a light microscope at × 40 magnification. The scale was similar to that used to assess the radiographic images. A carious lesion was defined as a demineralized, opaque white, or yellowish brown discolored area in the enamel or dentin (Figs. 1 and 2), and one result (one “truth”) of the histological examination was recorded.

### Data analysis

To simplify the comparison of the data, radiographic recordings were aggregated as follows: sound (score 0), enamel caries (score 1 + 2), and dentin caries (score 3 + 4). The scores were compared with the histological gold standard using receiver operating characteristic (ROC) curve analysis to assess the diagnostic accuracy of the presence or absence of caries. Areas under the ROC curve (Az) for each image type were compared for each image type using the Analyse-it program, Microsoft Exel, Method Evaluation Edition, and Statistica 10 programs. The values obtained for areas under the ROC curve were compared in pairs, with a significance level of 0.05.

Analyses were performed for all noncavitated lesions and separately for occlusal and approximal carious lesions.

## Results

Histological examination of 270 approximal surfaces revealed no caries on 157 surfaces (58.1 %), carious lesions extending into the outer half of the enamel on 51 surfaces (18.9 %), carious lesions extending into the inner half of the enamel on 23 surfaces (8.5 %), carious lesions extending into the outer third of the dentin on 25 surfaces (9.3 %), and deeper dentinal carious lesions on 14 surfaces (5.2 %).

The actual status of the 135 occlusal surfaces, as determined by histological examination, was as follows: 15 sound (11.11 %), 26 (19.26 %) with caries lesions extending into the outer half of the enamel, 41 (30.37 %) with caries extending into the inner half of the enamel, 33 (24.44 %) with caries in the external third of the dentine, and 20 (14.81 %) with deeper dentinal carious lesions.

The areas under the ROC curve (Az) and their standard errors are presented in Table 1 for approximal surfaces and for occlusal surfaces.

**Table 1:** Az values, 95 % confidence interval (CI), and standard errors (SE) of approximal surfaces and occlusal surfaces

	Test	Az	95 % CI	SE
Approximal surfaces	CBCT	0.629	0.580–0.679	0.025
	Digora	0.665	0.616–0.713	0.025
	Film	0.667	0.619–0.716	0.025
Occlusal surfaces	CBCT	0.635	0.547–0.722	0.045
	Digora	0.581	0.485–0.677	0.049
	Film	0.613	0.522–0.703	0.046

The lowest Az values were obtained with CBCT for approximal surfaces, and there were statistically significant differences between the Az values for CBCT and the PSP plate (*p* < 0.03) and between the Az values for CBCT and for intraoral film (*p* < 0.01).

CBCT had significantly higher Az values than the PSP plate (*p* < 0.05), but there were no statistically significant differences between the Az values for CBCT and film (*p* > 0.33) for occlusal surfaces (Table 2).

**Table 2:** Pairwise comparison of approximal surfaces and occlusal surfaces

		Difference between areas	95 % CI	SD	Z statistics	*p* Value
Approximal surfaces	CBCT/digora	0.0351	0.0024–0.0677	0.0167	2.1066	0.0352
	CBCT/film	0.0380	0.0088–0.0672	0.0149	2.5499	0.0108
	digora/film	0.0029	−0.0267–0.0326	0.0151	0.1938	0.8463
Occlusal surfaces	CBCT/digora	0.0535	−0.0993– − 0.0077	0.0234	−2.2877	0.0222
	CBCT/film	−0.0222	−0.0674–0.0229	0.0230	−0.9647	0.3347
	Digora/film	0.0313	0.0139–0.0486	0.0089	3.5221	0.0004

**Fig. 3:**
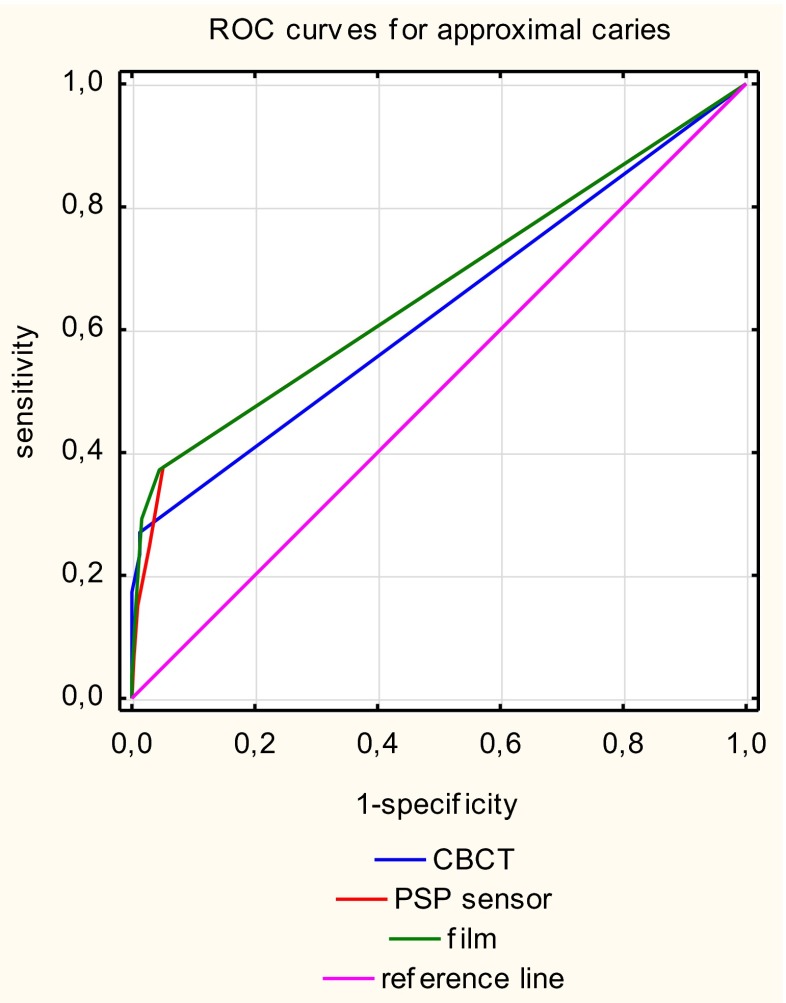
Receiver operating curves for each caries detection methods for approximal surfaces

**Fig. 4:**
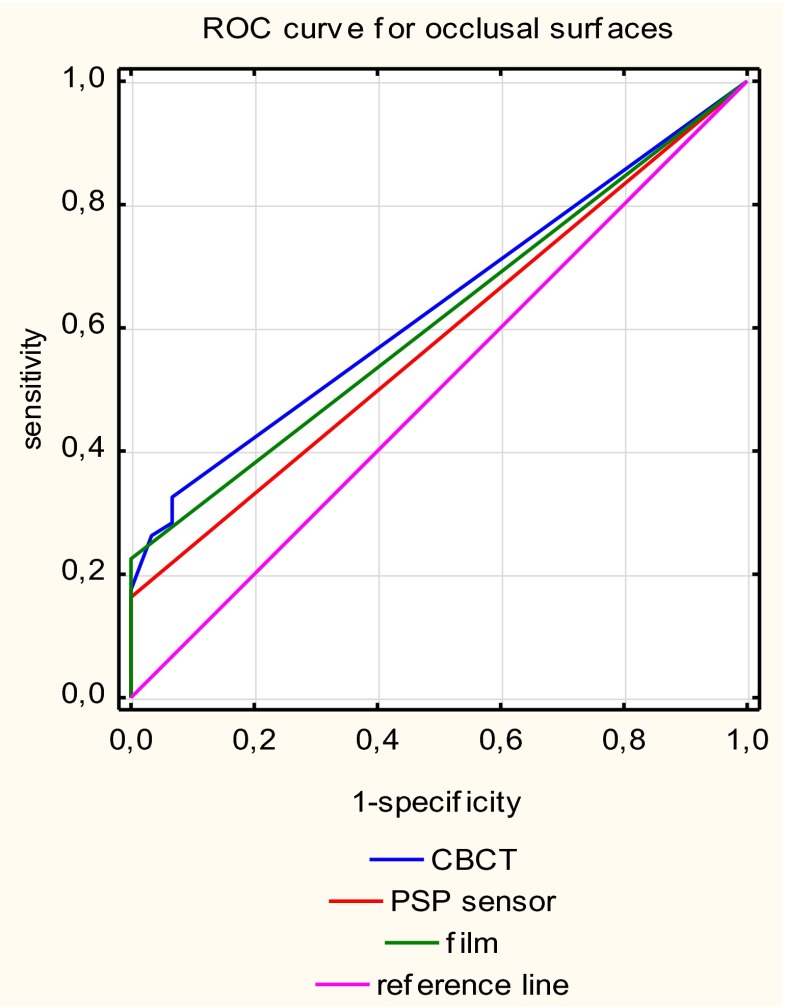
Receiver operating curves for each caries detection methods for occlusal surfaces

Figures 3 and 4 show the ROC curves of each imaging modality for the first reading of approximal and occlusal surfaces, respectively.

## Discussion

In the present study, only teeth with no cavitation of either approximal or occlusal surfaces were investigated. The surfaces of the teeth selected for the study mostly had incipient or superficial caries.

For approximal surfaces, the highest Az values were obtained from the PSP plate and conventional film. The differences between the Az for the CBCT system and for both intraoral modalities were significant. This indicates a lower diagnostic value of NewTom 3G. Similar result from a study using this CBCT system can be found in the study of Haiter-Neto et al. [17]. The authors examined performance of 3DX Accuitomo, NewTom 3G CBCT with different FOVs (6, 9 and 12 in) and two intraoral radiographic modalities (Kodak Insight film and Digora-Fmx digital system) in the detection of approximal and occlusal caries. However all the systems they used were evaluated using sensitivity and specificity parameters. They concluded that the 3G NewTom system had a lower diagnostic accuracy for the detection of caries than the intraoral modalities.

These results could be explained by the type of detector used in the investigation. This CBCT unit type is fitted with an IIT/CCD (image intensifier tube/charge-coupled device). It has been reported that, in comparison with flat panel detector systems, IIT/CCD devices have increased pixel noise and number of image artifacts [26]. Moreover, the disadvantages of CBCT include low spatial resolution and contrast [27]. The spatial resolution of CBCT is about 2 l p/mm [28, 29], while that for spatial resolution of conventional film is about 20 l p/mm [30]. This may explain why subtle changes in the degree of mineralization of tissues (including enamel) are impossible to distinguish from surrounding intact structures. Among artifacts that make the detection of caries more difficult are artifacts related to beam hardening which results in two types of artifacts: the distortion caused by metallic structures (known as cupping artifacts) and streaks and dark bands. The limitations of this study are the in vitro conditions employed and the selection of materials. Teeth with any fillings were excluded. In a true clinical situation, various fillings or other metallic elements (such as implants, orthodontic/dental braces, and prosthetic restorations) could be present in the patient’s mouth. These materials as well as possible patient movement during the examination could be responsible for the formation of errors in the resulting CBCT data.

Tsuchida et al. [16] tested the ability of a CBCT system to detect proximal caries. Using a CBVI system, namely, the three-dimensional Accuitomo unit, they reached an Az of 0.63 ± 0.02. This unit also employed a detector of the IIT and CCD type. Their analysis, however, showed no statistically significant detection differences between three-dimensional Accuitomo and Kodak Insight film.

Qu et al. [22] compared the use of five CBCT systems, operating with different detectors, in the detection of noncavitated proximal carious lesions. Two of these systems employed an IIT/CCD detector (NewTom 9000 and Accuitomo 3DX), and three systems employed flat panel detectors composed of amorphous silicon (Kodak 9000 3D) or CMOS (ProMax 3D and DCT PRO). The average Az values were very low, ranging from 0.541 to 0.577, and the authors found no statistically significant differences in the detection of approximal caries in the five systems. They concluded that the detector type employed in CBCT systems has no effect on the accuracy of approximal caries diagnosis.

Also, Zhang et al. [21] found no statistically significant differences when using either of the two CBCT systems (ProMax 3D and Kodak 9000 3D), *E* speed film or a PSP imaging system (Digora Optima). This confirms the results of previous studies by Tsuchida et al. [16] and Qu et al. [22]. Moreover, the Az values obtained were near the discriminative ability of the test. However, the diagnostic ability of CBCT in proximal caries detection could be higher when the caries extends into dentin [18, 19].

When considering occlusal carious lesions, the only statistically significant difference found in the present study was that between the Az for CBCT and that for a PSP plate. Higher Az values were obtained with NewTom 3G images. However, no statistical difference was found between CBCT and conventional film. However, some studies have reported greater diagnostic values of CBCT examination than with the other intraoral modalities for determining the presence of occlusal caries [17, 18, 23]. These results vary with the type of CBCT system used. Accuitomo had significantly higher sensitivity scores for the detection of dentinal lesions in approximal and occlusal surfaces than film and Digora. The NewTom 3G machine had the worst score [17] for detecting occlusal lesions limited to enamel. Young et al. [18] reported similarly low scores for occlusal lesions extending into dentin. The Kodak 9500 Cone Beam 3D system obtained statistically significantly higher diagnostic accuracy (using Az scores) in the detection of cavitated and noncavitated carious lesions in occlusal surfaces compared with Ektaspeed film and a storage PSP system (Digora Optime) [23].

## Conclusion

The accuracy of detection of noncavitated proximal caries with a NewTom 3G 9″ unit using Az analysis was significantly worse than that with the other intraoral radiographic modalities tested. However, the accuracy of the NewTom 3G 9″ system was slightly better for detecting noncavitated occlusal carious lesions than other intraoral radiographic modalities, but statistically significant differences were only obtained between the CBCT system and digital radiography. The diagnostic accuracy of the three radiographic systems tested is not sufficient, and when incipient or superficial carious lesions are suspected in either approximal or occlusal surfaces, it is necessary to use other methods to improve detection.

## References

[1] Pitts NB (2004). Are we ready to move from operative to non-operative/preventive treatment of dental caries in clinical practice?. Caries Res.

[2] Ismail AI (2006). Clinical diagnosis of precavitated carious lesions. Community Dent Oral Epidemiol.

[3] Pitts NB (1997). Diagnostic tools and measurements—impact on appropriate care. Community Dent Oral Epidemiol.

[4] Pitts NB (1996). The use of bitewing radiographs in the management of dental caries: scientific and practical considerations. Dentomaxillofac Radiol.

[5] Wenzel A (1995). Current trends in radiographic caries imaging. Oral Surg Oral Med Oral Pathol Oral Radiol Endod.

[6] van der Stelt PF (2008). Better imaging: the advantages of digital radiography. J Am Dent Assoc.

[7] Sukovic P (2003). Cone beam computed tomography in craniofacial imaging. Orthod Craniofacial Res.

[8] Farman AG, Scarfe WC (2009). The Basics of maxillofacial cone beam computed tomography. Semin Orthod.

[9] Ziegler CM, Woertche R, Brief J, Hassfeld S (2002). Clinical indications for digital volume tomography in oral and maxillofacial surgery. Dentomaxillofac Radiol.

[10] Rugani P, Kirnbauer B, Arnetzl GV, Jakse N (2009). Cone beam computerized tomography: basics for digital planning in oral surgery and implantology. Int J Comput Dent.

[11] Kau CH, Richmond S, Palomo JM, Hans MG (2005). Three-dimensional cone beam computerized tomography in orthodontics. J Orthod.

[12] Patel S, Dawood A, Ford TP, Whaites E (2007). The potential applications of cone beam computed tomography in the management of endodontic problems. Int Endod J.

[13] Mol A, Balasundaram A (2008). In vitro cone beam computed tomography imaging of periodontal bone. Dentomaxillofac Radiol.

[14] Barghan S, Merrill R, Tetradis S (2010). Cone beam computed tomography imaging in the evaluation of the temporomandibular joint. J Calif Dent Assoc.

[15] Akdeniz BG, Gröndahl HG, Magnusson B (2006). Accuracy of proximal caries depth measurements: comparison between limited cone beam computed tomography, storage phosphor and film radiography. Caries Res.

[16] Tsuchida R, Araki K, Okano T (2007). Evaluation of a limited cone-beam volumetric imaging system: comparison with film radiography in detecting incipient proximal caries. Oral Surg Oral Med Oral Pathol Oral Radiol Endod.

[17] Haiter-Neto F, Dos Anjos Pontual A, Frydenberg M, Wenzel A (2007). A comparison of older and newer versions of intraoral digital radiography systems: diagnosing noncavitated proximal carious lesions. J Am Dent Assoc.

[18] Young SM, Lee JT, Hodges RJ, Chang TL, Elashoff DA, White SC (2009). A comparative study of high-resolution cone beam computed tomography and charge-coupled device sensors for detecting caries. Dentomaxillofac Radiol.

[19] Şenel B, Kamburoglu K, Uçok O, Yüksel SP, Ozen T, Avsever H (2010). Diagnostic accuracy of different imaging modalities in detection of proximal caries. Dentomaxillofac Radiol.

[20] Cheng JG, Zhang ZL, Wang XY, Zhang ZY, Ma XC, Li G (2012). Detection accuracy of proximal caries by phosphor plate and cone-beam computerized tomography images scanned with different resolutions. Clin Oral Investig.

[21] Zhang Z, Qu X, Li G, Zhang Z, Ma X (2011). The detection accuracies for proximal caries by cone-beam computerized tomography, film, and phosphor plates. Oral Surg Oral Med Oral Pathol Oral Radiol Endod.

[22] Qu XM, Li G, Zhang ZY, Ma X (2011). Detection accuracy of in vitro approximal caries by cone beam computed tomography images. Eur J Radiol.

[23] Kayipmaz S, Sezgin ÖS, Saricaoğlu ST, Çan G (2011). An in vitro comparison of diagnostic abilities of conventional radiography, storage phosphor, and cone beam computed tomography to determine occlusal and approximal caries. Eur J Radiol.

[24] Kamburoğlu K, Murat S, Yüksel SP, Cebeci ARI, Paksoy CS (2010). Occlusal caries detection by using a cone-beam CT with different voxel resolutions and a digital intraoral sensor. Oral Surg Oral Med Oral Pathol Oral Radiol Endod.

[25] Rathore S, Tyndall D, Wright J, Everett E (2012). Ex vivo comparison of Galileos cone beam CT and intraoral radiographs in detecting occlusal caries. Dentomaxillofac Radiol.

[26] Naitoh M, Hirukawa A, Katsumata A, Saburi K, Okumura S, Ariji E (2006). Imaging artifact and exposure conditions in limited-volume cone-beam computed tomography: comparison between an image intensifier system and a flat panel detector. Oral Radiol.

[27] Katsumata A, Hirukawa A, Noujeim M, Okumura S, Naitoh M, Fujishita M (2006). Image artifacts in dental cone-beam CT. Oral Surg Oral Med Oral Pathol Oral Radiol Endod.

[28] Arai Y, Tammisalo E, Iwai K, Hashimoto K, Shinoda K (1999). Development of a compact computed tomographic apparatus for dental use. Dentomaxillofac Radiol.

[29] Gupta R, Cheung AC, Bartling SH, Lisauskas J, Grasruck M, Leidecker C (2008). Flat-panel volume CT: fundamental principles, technology, and applications. Radiographics.

[30] Farman AG, Farman TT (2005). A comparison of 18 different x-ray detectors currently used in dentistry. Oral Surg Oral Med Oral Pathol Oral Radiol Endod.

